# Intraoperative Neurophysiological Monitoring During Trigeminal Schwannoma Surgery

**DOI:** 10.7759/cureus.10218

**Published:** 2020-09-03

**Authors:** Faisal R Jahangiri, Abeera Azam, Rabehah A Asdi, Imtiaz Ahmad, Shaik I Basha

**Affiliations:** 1 Neurophysiology, Axis Neuromonitoring, Richardson, USA; 2 Neurophysiology, Global Innervation, Dallas, USA; 3 Behavioral and Brain Sciences, The University of Texas at Dallas, Richardson, USA; 4 Neuronavigation, King Abdulaziz Medical City, Ministry of National Guard-Health Affairs, Riyadh, SAU; 5 Ear, Nose, and Throat Surgery, Sheikh Khalifa Medical City, Abu Dhabi, ARE

**Keywords:** trigeminal schwannoma, trigeminal neuralgia, somatosensory evoked potentials, electromyography, electroencephalography, ionm, ssep, emg, trigeminal nerve.

## Abstract

Surgical manipulation during skull base surgeries places various cranial nerves (CN) at risk, including the nerves innervating the extraocular muscles. It could be very challenging for the surgeon to identify these cranial nerves due to the distortion of the normal anatomy by the tumors. Despite the recent advancement in technology, surgeries involving the third, fourth, fifth, and sixth cranial nerves still carry a risk of temporary or permanent paralysis of the muscles supplied by these cranial nerves. Intraoperative Neurophysiological Monitoring (IONM) with spontaneous and triggered electromyography (EMG) can help in guiding the surgeon in locating the nerves and avoiding any injury to them during the resection. IONM for extraocular cranial nerves requires highly skilled personnel with knowledge of anatomy and expertise in the placement of the electrodes. Benign tumors of the nerve sheath that arise from the perineural Schwann cells are known as schwannomas. Various cranial nerves might be involved in schwannomas of the head and neck. Trigeminal schwannomas are rare tumors. In this report, we describe the setup and stimulation technique and parameters as well as the benefits of utilizing IONM during the aggressive resection of a trigeminal schwannoma. The main purpose of utilizing IONM during these high-risk surgical procedures is to minimize any intraoperative damage to the neural structures involved.

## Introduction

Cranial nerves (CN) are at risk of injury during surgeries performed around them. Oculomotor nerve (CN III) injury can be caused by the insufficient blood supply and compression caused by aneurysm and tumor. CN III palsy results in diplopia, droopy eyelid, and enlarged pupil. It can also lead to the paresis of eye adduction and downward/upward gaze. The trochlear nerve (CN IV) can be damaged in the skull base, brainstem, and cavernous sinus surgeries. CN IV palsy can lead to vertical diplopia with weakness of downward eye movement. The affected eye shifts upward to the normal eye. The leading causes of CN IV palsy are head trauma (53%), followed by surgery (14%), and inflammation (14%) [1]. Abducens nerve (CN VI) can be damaged during skull base, cavernous sinus, and brainstem surgeries. Abducens palsy has been reported in 16-50% of patients undergoing skull base surgeries [2]. Abducens palsy leads to diplopia and inability to abduct eyes. Very few studies have documented the advantage of CN VI monitoring in surgery. Kaspera et al. have reported a statistically significant difference in the correct identification of CN VI (70% vs. 23%) with Intraoperative Neurophysiological Monitoring (IONM) during surgery of cavernous sinus meningiomas [3]. IONM of CN VI has also been used in brainstem surgery and midbrain surgery [4].

## Case presentation

**Figure 1:**
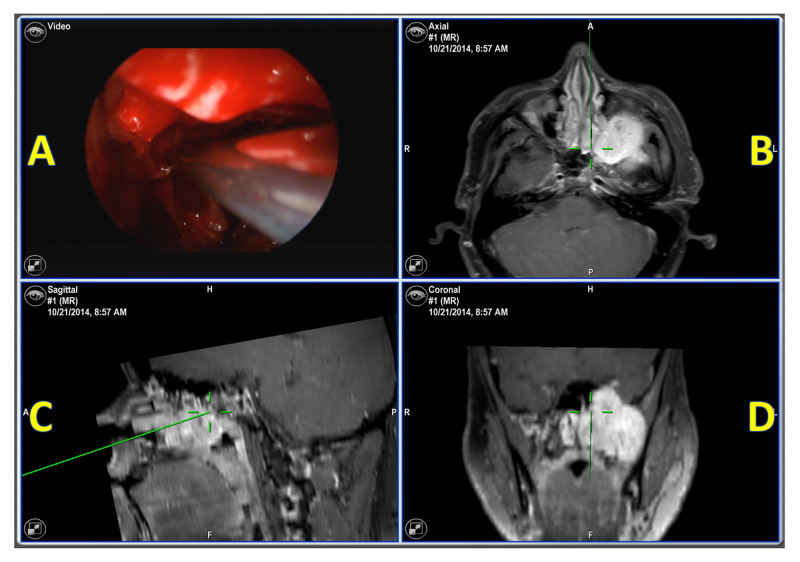
Intraoperative neuronavigation Intraoperative neuronavigation showing a large left sinus tumor. (A) endoscopic video source image; (B) axillary view at the maxillary sinus; (C) sagittal view at the maxillary sinus; (D) coronal view at the maxillary sinus

A 28-year-old male patient presented with a large left trigeminal schwannoma. MRI showed a large left sinus tumor involving multiple cranial nerves (Figure 1). The surgeon planned to do a craniotomy with a gross total resection. Total intravenous anesthesia (TIVA) with propofol and remifentanil infusion was used for anesthesia. Multimodality IONM setup was planned with somatosensory evoked potentials (SSEP), spontaneous electromyography (sEMG), triggered electromyography (tEMG), and electroencephalography (EEG).

**Figure 2:**
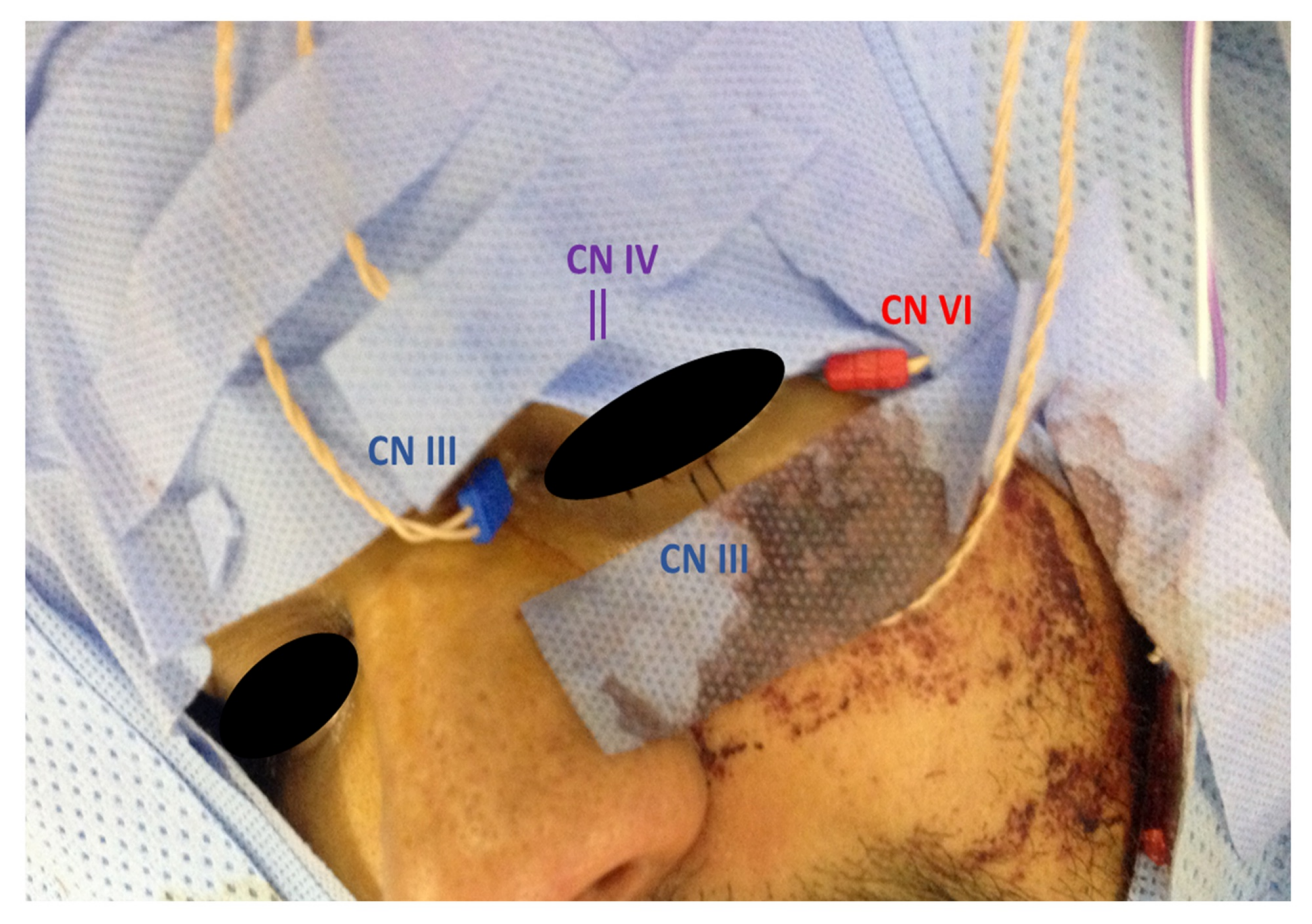
Patient setup The subdermal needle electrodes placed for electromyography recordings for cranial nerve III (oculomotor) in the medial rectus and inferior rectus (blue), for cranial nerve IV (trochlear) in the superior oblique (purple), and for cranial nerve VI (abducens) in lateral rectus muscles (red)

After patient intubation, surface adhesive electrodes were placed for stimulating ulnar SSEP in upper extremities and posterior tibial nerve SSEP in lower extremities. The subdermal needle electrodes were placed at popliteal fossa, Erb's point, fifth cervical vertebra (CV5), FPz, CPz CP3, and CP4, according to the international 10-20 system. The subdermal needle electrodes were placed for EMG recordings for CN III (oculomotor) in the medial rectus, for CN IV (trochlear) in the superior oblique, and for CN VI (abducens) in lateral rectus muscles (Figure 2).

**Figure 3:**
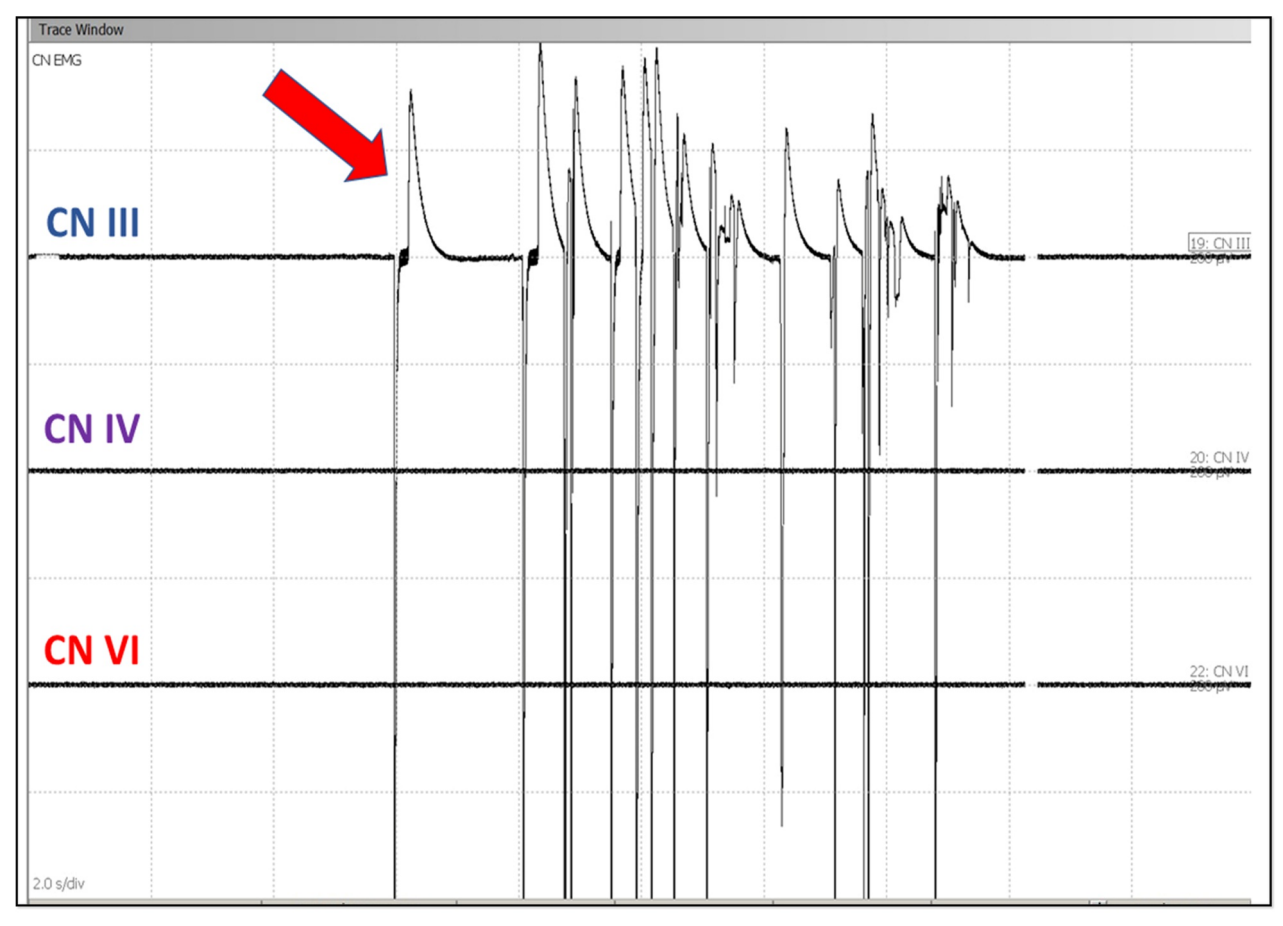
Spontaneous electromyogram Spontaneous electromyogram responses showing EMG train activity in cranial nerve III from medial rectus muscle (red arrow) due to nerve irritation CN III: oculomotor nerve; CN IV: trochlear nerve; CN VI: abducens nerve

The surgeon was informed of good baseline SSEP and EMG responses and was also continuously updated about the responses. sEMG train activity was reported to the surgeon during the tumor resection. During and after tumor resection, the surgeon performed triggered tEMG to identify the cranial nerves by using a monopolar fine tip probe. tEMG responses were recorded at 0.5 mA by direct monopolar nerve stimulation (Figure 3).

IONM was performed by a Certification in Intraoperative Neurophysiological Monitoring (CNIM)-certified [5] technologist and a Diplomate American Board of Neurophysiologic Monitoring (D.ABNM) board-certified [6] neurophysiologist. A board-certified neurologist with a specialty in IONM was also present online for remote monitoring during the entire surgical procedure [7]. In this patient, the multimodality IONM helped in guiding the surgeon during the high-risk procedure. Successfully monitored during the tumor resection, the patient woke up with no postoperative neurological deficit. The tumor attached to the cranial nerves was resected without any functional loss intraoperatively.

Postoperatively, the patient's neurological examination showed no neurological deficits involving cranial nerves III, IV, and VI at 24-hour, one-week, and three-month follow-ups.

## Discussion

Cranial nerves can be located within and around the tumor for cranial nerve mapping (CNM). Surgeons typically use anatomical landmarks to localize the cranial nerves. Depending on the location of the tumor, these anatomical landmarks are usually disturbed and shifted in various directions. The cranial nerves can be identified and tracked in the exposed area before, during, and after the tumor resection by a hand-held monopolar or bipolar stimulator with a constant current stimulator. Even though intraoperative CNM can help to prevent any damage to cranial nerves during the surgeries, there are some limitations because it is not continuous monitoring. Damage cannot be prevented if it occurs during the tumor resection without CNM. The CNM will also not identify any damage from the motor cortex to the muscles supplied by the cranial nerves proximal to the site of stimulation. Corticobulbar motor evoked potentials (Co-MEP) can be utilized intraoperatively for monitoring and preventing any damage to the motor pathways (corticobulbar tracts) [8]. A multimodality approach by combining CNM with sEMG, tEMG, and MEP can help in guiding the surgeon for resecting trigeminal schwannomas without causing any postoperative neurological deficits. SSEP should also be used for monitoring for cortical and brainstem ischemia. Muscles used for CNM are the medial/inferior rectus (CN III), superior oblique (CN IV), and lateral rectus (CN VI).

The location of the cranial nerves can be identified by stimulating with a monopolar or bipolar concentric hand-held probe. The stimulation probe should be moved a few millimeters until the responses are recorded by tEMG. The main goal of utilizing IONM is to protect the neural structures at risk and minimize any postoperative neurological deficits. Cranial nerves such as oculomotor, trochlear, trigeminal, and abducens are at risk. Electrooculography (EOG) is another reported technique for intraoperative monitoring of extraocular motor nerves (oculomotor and abducent nerves) by their extraocular movement (EOM). EOG was found to be a reliable and simple method, but quantitative data cannot be developed from that [9].

Utilizing the SSEP, EEG, sEMG, and tEMG during manipulation and tumor resection helps in identifying any pressure or stretch on the cranial nerves. Intraoperative use of sEMG and tEMG during the surgical procedures for the resection of the trigeminal schwannoma can be highly beneficial to the patient as well as the surgeon [10,11]. We recommend including Co-MEP for future procedures. The addition of Co-MEP will help in monitoring the corticobulbar motor pathways of the cranial nerves. Real-time feedback to the surgeon about the oculomotor, trochlear, and abducens nerves can minimize any postoperative neurological deficits involving the motor function of the eye [12,13].

## Conclusions

IONM of the cranial nerves innervating the extraocular muscles is highly beneficial in preventing any injuries to these nerves intraoperatively. Trigeminal schwannoma resection can put various cranial nerves at risk due to distortion of the normal anatomical landmarks. In this patient, the sEMG and tEMG were used effectively for the prevention of any loss of eye motor function intraoperatively by identifying the cranial nerves involved by stimulating the cranial nerves during the tumor resection. The neurophysiological monitoring utilizing EMG helped in directing the surgeon intraoperatively. During surgeries that put the cranial motor nerves at risk of injury, continuous monitoring of the function by EMG by accurate stimulation and recording parameters is desirable.
